# Determination of Propionylbrassinolide and Its Impurities by High-Performance Liquid Chromatography with Evaporative Light Scattering Detection

**DOI:** 10.3390/molecules23030531

**Published:** 2018-02-27

**Authors:** Lidong Cao, Hong Zhang, Hongjun Zhang, Li Yang, Miaomiao Wu, Puguo Zhou, Qiliang Huang

**Affiliations:** 1 Institute of Plant Protection, Chinese Academy of Agricultural Sciences, No. 2 Yuanmingyuan West Road, Beijing 100193, China; caolidong@caas.cn (L.C.); hongapplezh@163.com (H.Z.); huaweimian666666@163.com (L.Y.); wumiaomiao2016@163.com (M.W.); 2 Institute for the Control of Agrochemicals, Ministry of Agriculture, No. 22 Maizidian Street, Beijing 110000, China; hongjun-zh1975@163.com

**Keywords:** propionylbrassinolide, impurities, HPLC-ELSD, mass spectrometry, method validation

## Abstract

The discovery of brassinolide in 1979, a milestone in brassinosteroids research, has sparked great interest of brassinolide analogs (BLs) in agricultural applications. Among these BLs, propionylbrassinolide has captured considerable attention because it shows plant growth regulating activity with an excellent durability. Two impurities of propionylbrassinolide were isolated and purified by semi-preparative high-performance liquid chromatography (HPLC), and the chemical structures were confirmed. For simultaneous separation and determination of propionylbrassinolide and impurities, an efficient analytical method based on HPLC with evaporative light scattering detector (HPLC-ELSD) was developed. The optimized analysis was performed on a C18 reversed phase column (250 mm × 4.60 mm, 5 μm) with isocratic elution of acetonitrile and water (90:10, *v*/*v*) as the mobile phase. The drift tube temperature of the ELSD system was set to 50 °C and the auxiliary gas pressure was 150 kPa. The regression equations demonstrated a good linear relationship (*R*^2^ = 0.9989–0.9999) within the test ranges. The limits of detection (LODs) and quantification (LOQs) for propionylbrassinolide, impurity 1 and 2 were 1.3, 1.2, 1,3 and 4.3, 4.0, 4.2 mg/L, respectively. The fully validated HPLC-ELSD method was readily applied to quantify the active ingredient and impurities in propionylbrassinolide technical concentrate. Moreover, the optimized separation conditions with ELSD have been successfully transferred to mass spectrometry (MS) detector for LC-MS determination.

## 1. Introduction

**Figure 1:**
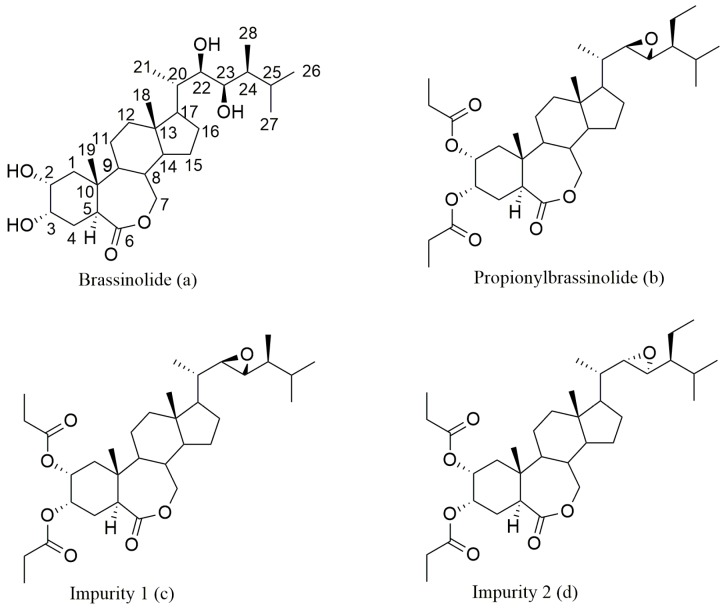
Chemical structures of the brassinolide, propionylbrassinolide and its impurities.

Brassinosteroids (BRs), a class of triterpenoid polyhydroxy endogenous steroid phytohormones, are ubiquitously distributed in the plant kingdom [1,2]. BRs, viewed as the sixth phytohormones in addition to auxins, gibberellins, cytokinins, abscisic acid and ethylene, regulate a variety of biological processes of plants even at very low concentrations [3]. The importance of BRs in plant growth and development has sparked great interest in agricultural applications [4], even reduce the pesticide residues [5]. The isolation and structure identification of brassinolide [22*R*,23*R*,24*S*–2α,3α,22,23-tetrahydroxy-24-methyl-B-homo-7-oxa-5α-cholestan-6-one (Figure 1a)] by Grove et al. in 1979 was a milestone in BRs research [6]. It opened a new horizon for researchers to study plant growth hormones. Since then, different plant phyla were screened for the presence of these steroidal compounds. However, the extreme low abundances of brassinolide and other BRs in natural plants impose restrictions on their academic research and practical applications. The chemical synthesis of brassinolide analogs (BLs) from readily accessible starting materials (ergosterol and stigmasterol) has addressed such limitations. Among these BLs, propionylbrassinolide, [22*R*,23*R*,24*S*–2α,3α–dipropionyloxy-22,23-epoxy-B-homo-7-oxa-5α-cholestan-6-one (Figure 1b)], has captured great attention because it shows plant growth regulating activity with an excellent durability, which is ascribed to the slow release of 2α,3α-dihydroxy groups by hydrolysis [7]. In view of the potent bioactivity, it is imperative to develop an efficient analytical method for quality control of propionylbrassinolide as well as its impurities formed during the production process.

High-performance liquid chromatography (HPLC) frequently served as the method of choice for the separation and analysis of BLs in various plant matrices [8]. However, the absence of suitable chromophores brings about problems in their detection. Therefore, BLs are often derivatized with pre-labeling reagents to make them responsive to ultraviolet, fluorimetric, or electrochemical detectors [8,9]. For this purpose, diverse boronic acid derivatization reagents have been used to condensed with the vicinal diol groups in BLs to produce the corresponding boronates, which exhibit strong absorption to facilitate extraction and detection. The boronic acids reported mainly included naphthaleneboronic acid [10], 9-phenanthreneboronic acid [11], ferroceneboronic acid [12], dansyl-3-aminophenylboronic acid [13], 2-bromopyridine-5-boronic acid [14], quaternary ammonium phenyl boronic acid [15], 4-phenylaminomethylbenzeneboric acid [16], and magnetic phenylboronic acid nanoparticles [17]. Due to the sensitivity and speed of analysis, the cutting-edge mass spectrometry (MS)-based methods have also been applied frequently for the determination of BLs [8]. For facile propionylbrassinolide detection, a HPLC method based on precolumn boronic acid derivatization is impossible since the vicinal diol groups in C22 and C23 are replaced with epoxy group. Liu et al. developed a GC–MS method for residue analysis of propionylbrassinolide in fruit and vegetables [18]. Taking advantages of the merits of HPLC, efficient HPLC-based analytical procedures for separating and assaying propionylbrassinolide are still lacking.

Over the past four decades, evaporative light scattering detection (ELSD) has grown in popularity and moved into the mainstream of HPLC detection techniques [19,20]. This technique can detect any analyte that is less volatile than the mobile phase, regardless of the optical, electrochemical, or other properties. ELSD therefore has been widely used to detect compounds such as carbohydrates [21,22], glycosides [23,24], fatty acids [25], lipids [26], Chinese medicinal preparation [27], and surfactants [28] that possess no or only a weak UV chromophore. In view of the optical properties of BLs that have weak absorption even in the short wavelength range, BLs are indeed the suitable candidates for HPLC detection coupled with ELSD.

Recently, we reported a HPLC-ELSD method for the determination of four BLs including 24-epibrassinolide, 22, 23, 24-trisepibrassinolide, 28-homobrassinolide and 28-epihomobrassinolide [29]. In the present study, two impurities (Figure 1c,d) of propionylbrassinolide were separated and purified by semi-preparative HPLC, and the chemical structures were confirmed by ^1^H-NMR, ^13^C-NMR, distortionless enhancement by polarization transfer (DEPT) NMR, and MS. Subsequently, an efficient HPLC-ELSD method was established and fully validated for the simultaneous separation and determination of propionylbrassinolide and its impurities. Moreover, the developed method has been successfully applied for the analysis of commercial propionylbrassinolide technical material to show the practicability and facility for quality control.

## 2. Results and Discussion

### 2.1. Structure Identification of Impurities

During the synthesis of propionylbrassinolide TC, all the factors such as impurities from the starting materials, incomplete or side reactions, degradation of the active constituent, may result in impurities. Impurities may affect the quality of active ingredient or result in undesired adverse effect to human and environment. Therefore, it is imperative to determine the impurities qualitatively and quantitatively. Due to the low abundance of impurities in propionylbrassinolide TC, it′s difficult to use the conventional flash column chromatography on silica gel to isolate these impurities. In the present study, semi-preparative HPLC was used to separate and purify the impurities. Two major impurities were isolated and subjected to ^1^H-NMR, ^13^C-NMR (including DEPT90 and DEPT135), and MS analysis. The NMR spectra of propionylbrassinolide and two impurities were presented in Supplementary Information (Figures S1–S12). Based on the NMR and MS analysis as well as the synthetic technology of propionylbrassinolide, the chemical structures of the two impurities are depicted in Figure 1c,d.

### 2.2. Optimization of Chromatographic Conditions

**Figure 2:**
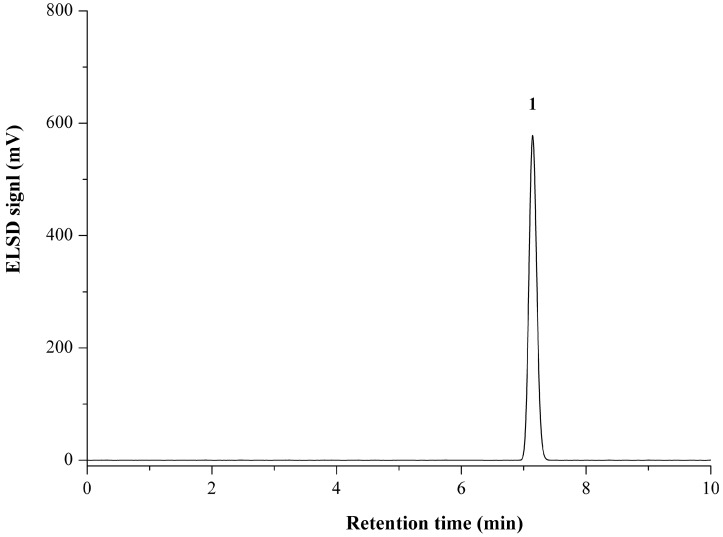
Typical high-performance liquid chromatography with evaporative light scattering detector (HPLC-ELSD) chromatogram of propionylbrassinolide standard on an Eclipse XDB-C_18_ column (250 mm × 4.6 mm, 5.0 μm) column.

**Figure 3:**
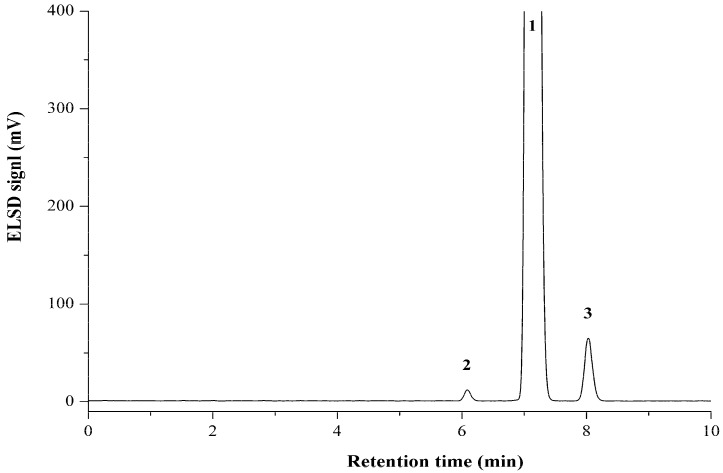
Typical HPLC-ELSD chromatogram of propionylbrassinolide technical concentrate on an Eclipse XDB-C_18_ column (250 mm × 4.6 mm, 5.0 μm) column. **1**: propionylbrassinolide; **2**: impurity 1; **3**: impurity 2.

The HPLC optimization was performed with the mixed standard solutions. Screening of the mobile phase demonstrated that isocratic elution with an acetonitrile–water mixture (90:10, *v*/*v*) using the Eclipse XDB-C_18_ column (250 mm × 4.6 mm, 5.0 μm) could afford satisfactory baseline separation on the targeted analytes within 10 min. The optimized detector conditions were determined by comparing the peak area values and baseline. Detector optimization indicated that the drift tube temperature of 50 °C, gain value of 10, and the auxiliary gas pressure of 150 kPa could provide approving results. The representative HPLC-ELSD chromatograms for propionylbrassinolide standard and TC are shown in Figure 2 and Figure 3.

### 2.3. Calibration and Validation

The linearity was evaluated by determination of five different concentrations of the standard solutions. The regression equations, LODs, and LOQs of the components are shown in Table 1. All calibration curves showed good linearity (*R*^2^ = 0.9989–0.9999) in the test range. The limits of detection (LODs) and quantification (LOQs) were in the range of 1.2–1.3 and 4.0–4.2 mg/L, respectively. Although the present HPLC-ELSD method is less sensitive than HPLC-MS method with the LOD of attomolar level by mean of 2-bromopyridine-5-boronic acid derivatization [14], its sensitivity is sufficient for efficient and quick determination of propionylbrassinolide TC and formulation without cutting-edge MS instrument, providing valuable information for quality control and process optimization. 

Precision was evaluated by measuring the mixed standard solution and real samples of propionylbrassinolide TC. For repeatability (intraday) and intermediate (interday) precision to be established, variations in the aspect of peak areas and retention times of the mixed standard solution were determined (Table 2). Under the repeatability conditions described in Section 2.4, retention times and peak areas of the tested compounds were steady with 0.5–0.8% and 2.9–4.6% RSD, respectively. While for intermediate (interday) precision, the counterparts were stable with 0.9–1.2% and 5.3–6.6% RSD, respectively, which are a little bit higher than what was found for intraday precision. The precision of developed method was also determined by comparing the variations among seven replicates measurements of the same batch of propionylbrassinolide TC with the Horwitz value (*%RSDr*). All the %RSD values of active ingredient and two impurities determinations were less than the corresponding *%RSDr* (Table 3), indicating that the developed method is precise. For method accuracy to be determined, the standard addition method was used. As demonstrated in Table 4, method accuracy was found to be satisfactory with the recoveries ranging from 92.03% to 99.07%. These validation results indicate that this HPLC-ELSD method is sensitive, precise, and accurate for the quantitative determination of propionylbrassinolide and its impurities.

### 2.4. LC-MS Analysis

**Figure 4:**
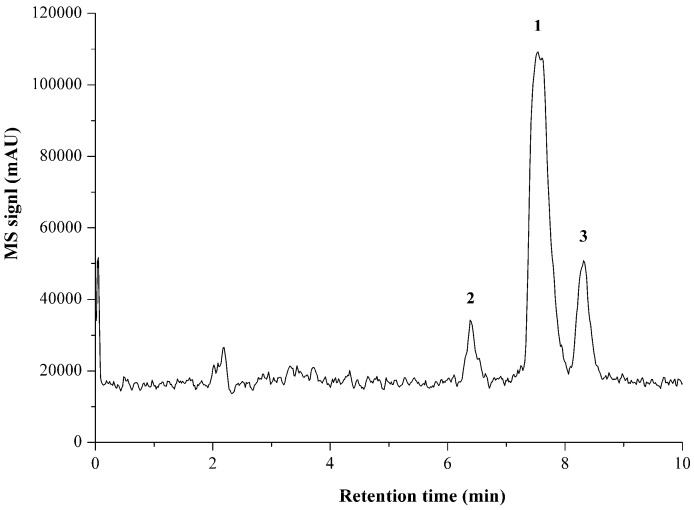
Representative LC-MS chromatogram of propionylbrassinolide TC under full scan model. **1**: propionylbrassinolide; **2**: impurity 1; **3**: impurity 2.

**Figure 5:**
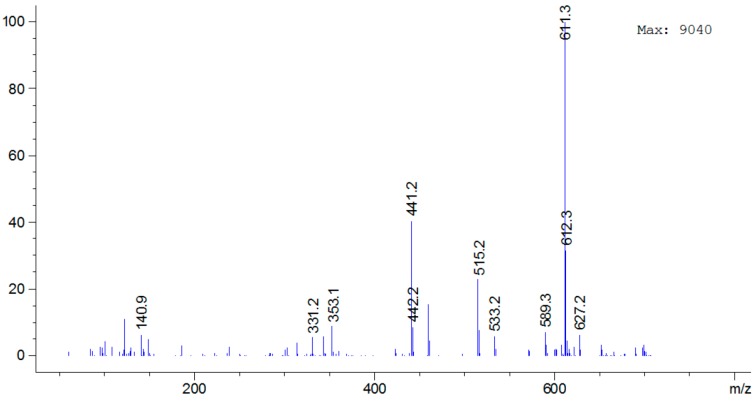
Mass spectrum of propionylbrassinolide recorded in the positive mode.

**Figure 6:**
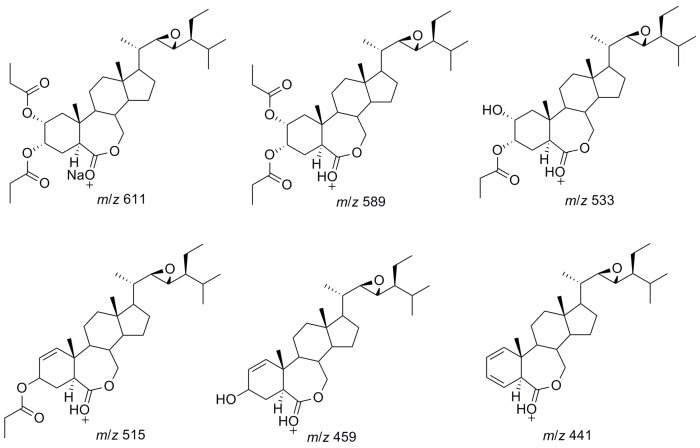
Proposed structure of the major mass ions for propionylbrassinolide.

For analysis of BLs, due to the non-volatile highly hydrophilic properties, LC-MS is generally the method of first choice. However, most of the methods require precolumn boronic acid derivatization [8], only several have so far been applied for the direct analysis of native BLs [30,31,32,33,34]. In the present investigation, the direct LC-MS determination of propionylbrassinolide and its impurities was for the first time to be demonstrated. The representative LC-MS chromatogram of propionylbrassinolide TC under full scan model is shown in Figure 4. Propionylbrassinolide and its two major impurities gave precise mass information, and the mass spectrum of propionylbrassinolide was shown in Figure 5. All the analytes of interest yielded stable quasi-molecular ions of [M + Na]^+^ (*m*/*z* of 611 for propionylbrassinolide and impurity 2, 597 for impurity 1) as the most abundant ion in the mass spectrum. This result is most possibly due to the strong attachment of sodium to the epoxy and ketone groups of steroids. However, in the GC-MS determination of propionylbrassinolide, molecular ion peak was not observed, and *m*/*z* of 329, 355, 459, and 503 derived from the fragmentation of the molecule were used as the characteristic product ions [18], which were different from those detected in LC-MS analysis due to the different ionization mode. The optimized GC-MS conditions reported by Liu et al. was as follows: the injector, ion source and transfer line were set at 300, 250 and 280 °C, respectively; the ion trap mass spectrometer was operated in the electron ionization mode at 50 eV. Due to the facile fragmentation of propionyl group, the fragmented ion peaks always exist in electrospray positive mode. The proposed structure of the major mass ions for propionylbrassinolide is presented in Figure 6. The mass spectra of the two propionylbrassinolide impurities as well as the proposed structures of the major mass ions are shown in Figures S13–S16. This developed LC-MS method can provide a quick and efficient qualitative determination for propionylbrassinolide and its impurities.

### 2.5. Method Application

Up to now, there is only one reported analytical method for propionylbrassinolide by GC-MS. It′s well accepted that the detection sensitivity is a critical factor for practical application of the established analytical method. Although the present HPLC-ELSD method is less sensitive than the GC-MS method with LODs and LOQs of 0.15 and 0.5 mg kg^−1^ in all samples [18], nevertheless, for quick, simple, and efficient quality control for propionylbrassinolide TC and formulations, the LODs are acceptable, and the facility as well as time-saving in experimental operation could enhance the practicability. To demonstrate the applicability of the established HPLC-ELSD method, commercial available propionylbrassinolide TC was determined. The active ingredient and two impurities are identified by comparison of the retention times with the standards, further verified by LC-MS. As shown in Table 3, the contents of propionylbrassinolide and two impurities were accurately quantified as 95.15%, 0.44% and 2.72%, respectively. The precision and accuracy determinations for active ingredient and impurities in propionylbrassinolide TC summarized in Table 3 and Table 4 indicate that the present HPLC-ELSD method was accurate and reliable. The present study further demonstrates that HPLC-ELSD method can detect any analyte that is less volatile than the mobile phase, regardless of the optical or electrochemical properties. Moreover, the impurities in pesticide TC may affect the quality of active ingredient or result in undesired adverse effect to human and environment. The present study further highlights the importance of determination of impurities.

## 3. Experimental

### 3.1. Reagents and Materials

HPLC-grade acetonitrile and methanol were purchased from Fisher Scientific (Fair Lawn, NJ, USA). Ultrapure water employed in all experiments was prepared using a MilliQ-50 SP reagent water system (Millipore Corporation, Bedford, MA, USA). Other reagents were of analytical grade. Analytical standards of propionylbrassinolide (Lab No. IPP-SD-711), impurity 1 (Lab No. IPP-SD-710) and impurity 2 (Lab No. IPP-SD-712) were purified by semi-preparative HPLC in our lab with purity more than 98% by peak area detected by HPLC-ELSD. The propionylbrassinolide technical concentrate (TC) with the purity of 95% was kindly provided by National Pesticides Quality Supervision and Inspection Center (Beijing, China). The stock solution of each standard was prepared in acetonitrile with approximate concentrations of 2000 mg/L. All the stock solutions were stored in the refrigerator at 4 °C until required for use. The working standard solutions were prepared as required by appropriate dilution of the concentrated stock solutions with acetonitrile.

0.02 g of propionylbrassinolide TC was accurately weighed and dissolved in 10 mL of acetonitrile to prepare stock sample solution, which was directly used for impurities determination. For propionylbrassinolide determination, the working sample solution was prepared by dilution of the stock sample solution to 200 mg/L with acetonitrile.

### 3.2. Semi-Preparative HPLC Conditions

The optimized semi-preparative HPLC operating conditions for propionylbrassinolide, impurities 1 and 2 were achieved on an Eclipse XDB-C_18_ column (250 mm × 21.2 mm, 7.0 μm) (Agilent, Santa Clara, CA, USA) with a mobile phase of methanol-water (*v*/*v*, 99:1), flow rate of 10 mL/min, injection volume of 800 μL, and detection wavelength at 210 nm.

### 3.3. HPLC-ELSD Analysis

HPLC-ELSD analysis was performed on an Agilent 1200 liquid chromatograph composed of the following modules: G1329A autosampler, G1316A column oven, G1311A quaternary pump, and G4218A ELS detector (Agilent technologies, Santa Clara, CA, USA) controlled by Agilent Chemstation LC Software. Separation was achieved on an Eclipse XDB-C_18_ column (250 mm × 4.6 mm, 5.0 μm) (Agilent, Santa Clara, CA, USA) at 30 °C. The mobile phase consisted of an acetonitrile-water mixture (90:10, *v*/*v*) was degassed by ultrasonication prior to use. Isocratic elution was carried out at a flow rate of 1.0 mL/min and 5 μL was injected. All samples and standards were filtered through 0.45 μm Millipore membrane before injection. The drift tube temperature for ELSD was set at 50 °C, gain value was 10, and the auxiliary nitrogen gas pressure was adjusted to 150 kPa.

### 3.4. Calibration

Linearity was evaluated using a linear regression analysis calculated by least square method. Calibration curves were constructed by plotting the logarithmic values of the peak area (ELSD signal) versus logarithmic values of the mass concentration (mg/L) of the analytes injected. The standard stock solutions were diluted to appropriate concentrations for construction of calibration curves. For linearity determination, seven levels concentrations were analyzed in duplicates. Narrower concentration range spanning 80–120% of the expected concentration in test item were analyzed in duplicates to construct the calibration curves, which were used for propionylbrassinolide and impurities determination in TC. The dilute standard solution was further diluted to the known low concentration with acetonitrile for signal-to-noise (S/N) ratio measurement. The limits of detection (LODs) and quantification (LOQs) were defined as the minimum concentrations giving S/N ratios of 3 and 10, respectively. 

### 3.5. Method Validation

Repeatability (intraday) and intermediate (interday) precision were used to determine the precision of the developed method and were expressed as relative standard deviation (%RSD). Repeatability in terms of retention time and peak area was validated seven times a day with a standard mixture solution. For interday precision, measurements (three times a day) on three consecutive days were conducted with the same equipment but performed by two testing personnel using three separately prepared batches of mobile phase. Moreover, the method precision was also assessed by the real sample determination. For test item of propionylbrassinolide TC, the contents of the active ingredient and two impurities were measured under the prescribed conditions. The coefficient of variations (CV) of seven replicates determinations of the same batch of TC are compared with the Horwitz value (*%RSDr*) [35]. The Horwitz equations are described as follows:

*%RSD_R_* = 2 ^(1 − 0.5 log^_10_^*C*)^ (1)

*%RSDr = %RSD_R_* × 0.67 (2)

where *%RSD_R_* represents the inter-laboratory CV, *%RSDr* represents the repeatability CV, and C represents the concentration of the analyte in the sample as a decimal fraction. 

The recovery test was used to evaluate the accuracy of this method. The recoveries were determined by standard addition method. A known quantity of individual working standard solution was added to a predetermined propionylbrassinolide TC, and the spiked sample was analyzed. The total amount of each analyte of interest was calculated from the corresponding calibration curve, and recovery of individual analyte was determined using the following formula: recovery (%) = (observed amount − original amount)/spiked amount × 100%. For each analyte, three levels addition were performed. Each determination was injected in duplicate.

### 3.6. LC-MS Analysis

The LC-MS analysis for qualitatively identification was carried out on an Agilent 1100 Series LC/MSD system (Agilent, Santa Clara, CA, USA) equipped with a single quadrupole mass detector and electrospray ionization (ESI) interface. The column and mobile phase composition are the same as in the HPLC-ELSD analysis, except that 0.1% formic acid aqueous solution was used instead of water. The mass spectrum was recorded in the positive mode with the scan range of *m*/*z* 50–800. Optimized mass conditions are as follows: drying gas (N_2_) flow rate of 3.0 L/min, temperature 300 °C, nebulizer pressure 275 kPa, capillary voltage 3500 V and fragmentor voltage 65 V.

### 3.7. NMR Analysis

^1^H and ^13^C-NMR data were obtained on a Bruker Avance 300 spectrometer operating at 300 MHz for ^1^H and 75 MHz for ^13^C using CDCl_3_ as solvent and TMS (tetramethylsilane) as the internal standard in 5-mm NMR tubes. Chemical shift data are reported in units of δ (ppm) according to the TMS signal. For structure identification, DEPT90 and DEPT135 ^13^C-NMR were also determined to provide the information of carbon types.

## 4. Conclusions

In this work, two impurities of propionylbrassinolide were obtained and purified by semi-preparative HPLC, and the chemical structures were confirmed by ^1^H-NMR, ^13^C-NMR, DEPT ^13^C-NMR, and MS. Subsequently, an efficient HPLC-ELSD method has been developed and demonstrated to be suitable to separate, identify, and quantify propionylbrassinolide and its two impurities within 10 min. The established method also showed satisfactory validation parameters in terms of sensitivity, linearity, accuracy, and precision, and can be readily applied for propionylbrassinolide TC determination with approving precision and accuracy. The optimized separation conditions with ELSD have been successfully transferred to mass detector for direct LC-MS determination for structure verification.

## Figures and Tables

**Table 1:** Linearity of calibration curve of propionylbrassinolide and two impurities.

Analyte	Linear Range (mg/L)	Calibration Curve *^a^*	*R*^2^	LOD (mg/L)	LOQ (mg/L)
Propionylbrassinolide	4.3–543.3	*y* = 1.5999*x*– 0.2860	0.9989	1.3	4.3
Impurity 1	4.0–502.3	*y* = 1.5912*x* – 0.3123	0.9997	1.2	4.0
Impurity 2	4.2–539.3	*y* = 1.6949*x* – 0.2952	0.9999	1.3	4.2

*^a^ y* and *x* refer to the logarithmic values of peak area and mass concentration (mg/L) injected. LOD: limit of detection; LOQ: limit of quantification.

**Table 2:** Determination of method precision under repeatability (intraday) and intermediate precision (interday) conditions reported as RSD (%) of peak area and retention time.

Analyte	Repeatability (*n* = 7)		Intermediate Precision (*n* = 9)	
	Peak Area	Retention Time	Peak Area	Retention Time
Propionylbrassinolide	4.64	0.53	6.64	0.87
Impurity 1	2.89	0.46	5.29	0.95
Impurity 2	3.56	0.76	6.42	1.22

**Table 3:** Determination of propionylbrassinolide and two impurities in propionylbrassinolide technical concentrate (TC).

Analyte	Mean Content (%, *n* = 7) *^a^*	%RSD	*%RSDr*
Propionylbrassinolide	95.15	0.51	1.35
Impurity 1	0.44	1.36	3.03
Impurity 2	2.72	1.85	2.30

*^a^* Content (%): Mass percentage of each composition in propionylbrassinolide TC.

**Table 4:** Accuracy determination of propionylbrassinolide and two impurities in propionylbrassinolide TC.

Analyte	Original (mg/L)	Spiked (mg/L)	Observed (mg/L)	Recovery (%)	Mean (%)	RSD (%)
Propionylbrassinolide	90.10	33.95	124.23	100.53	99.07	1.32
	125.30	33.95	158.58	98.03		
	170.28	33.95	203.77	98.65		
Impurity 1	3.81	3.93	7.26	87.79	92.03	4.70
	5.06	3.93	8.85	96.44		
	7.25	3.93	10.86	91.86		
Impurity 2	30.85	16.85	46.88	95.13	95.96	1.45
	39.24	16.85	55.28	95.19		
	52.21	16.85	68.65	97.57		

## Supplementary Materials

The following are available online.
