# Supplementary material for: Determination of Propionylbrassinolide and Its Impurities by High-Performance Liquid Chromatography with Evaporative Light Scattering Detection
Source: Molecules. 2018 Feb 27;23(3):531. doi: 10.3390/molecules23030531 (PMC6017011; doi:10.3390/molecules23030531)
Supplement: Supplementary file 1 [file molecules-23-00531-s001.pdf]

Supplementary Materials

Determination of Propionylbrassinolide and Its Impurities by High-Performance Liquid Chromatography with Evaporative Light Scattering Detection

Lidong Cao[1], Hong Zhang[1], Hongjun Zhang[2], Li Yang[1], Miaomiao Wu[1], Puguo Zhou[2,*], and Qiliang Huang[1,*]

[1] Institute of Plant Protection, Chinese Academy of Agricultural Sciences, No. 2 Yuanmingyuan West Road, Beijing 100193, China; caolidong@caas.cn (L.C.); hongapplezh@163.com (H.Z.); huaweimian666666@163.com (L.Y.) ; wumiaomiao2016@163.com (M. W.)

[2] Institute for the Control of Agrochemicals, Ministry of Agriculture, No. 22 Maizidian Street, Beijing 110000, China; hongjun-zh1975@163.com (H.Z.)

*Correspondence: zhoupuguo@sohu.com, Tel./Fax: +86 10 59195230; qlhuang@ippcaas.cn; Tel./Fax: (+86) 10 62816909

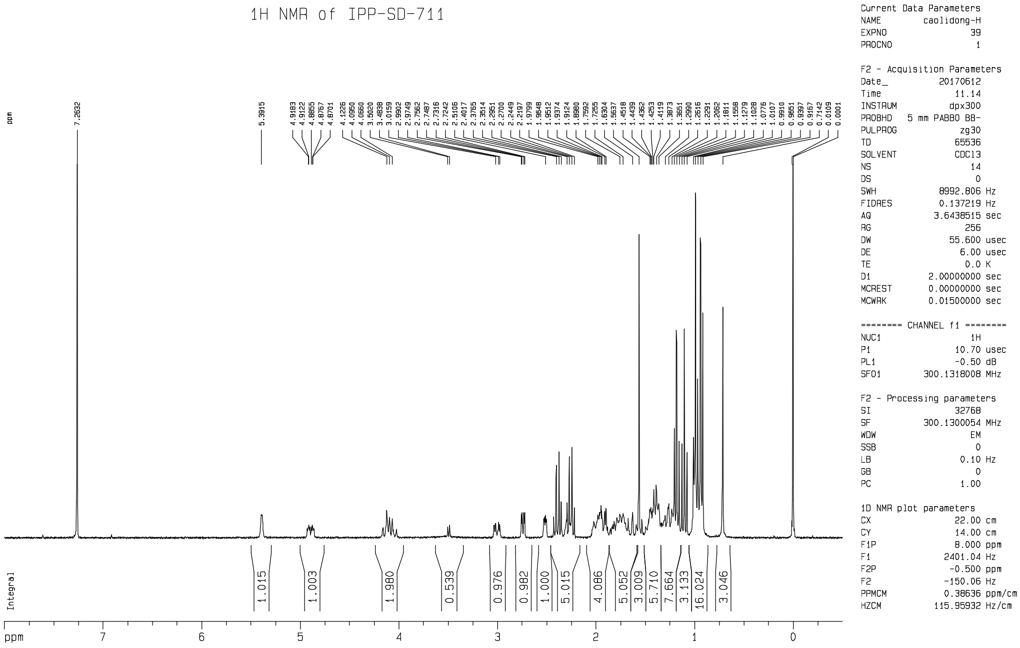

Figure S1. ^{1}H NMR of propionylbrassinolide

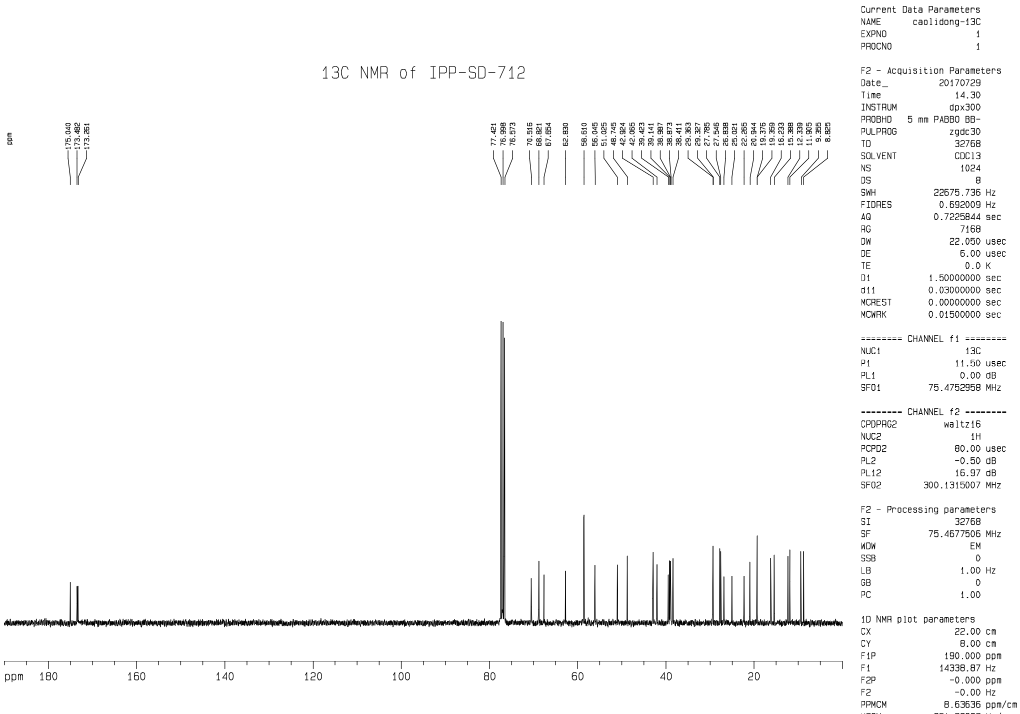

Figure S2. ^{13}C NMR of propionylbrassinolide

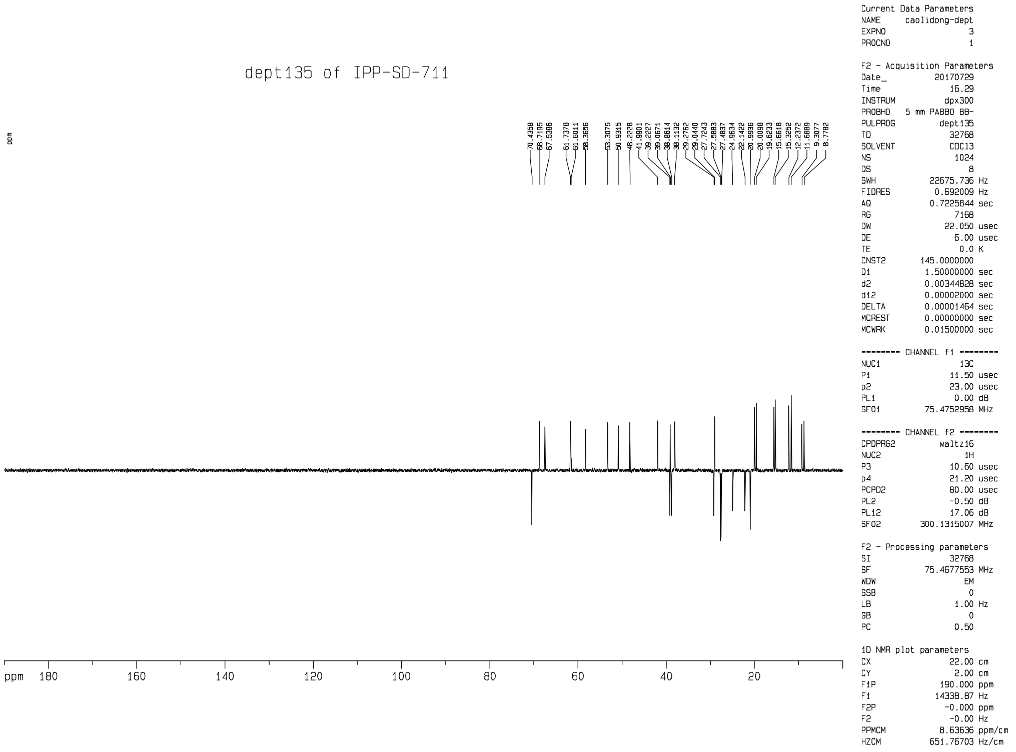

Figure S3. ^{13}C NMR (DEPT 135) of propionylbrassinolide

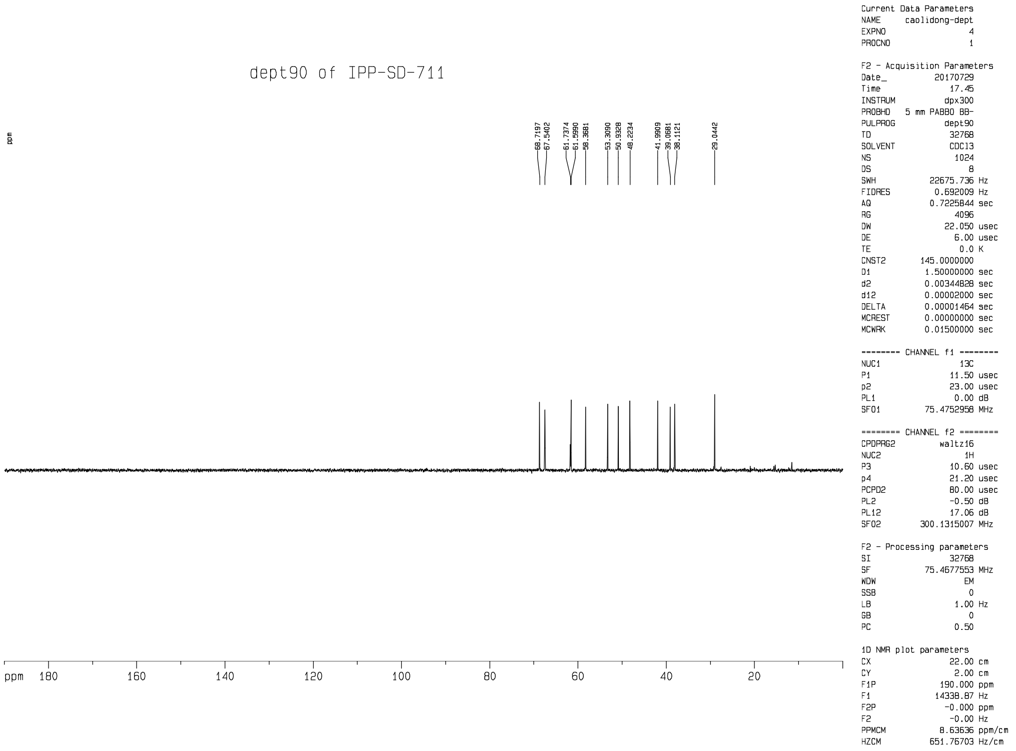

Figure S4. ^{13}C NMR (DEPT 90) of propionylbrassinolide

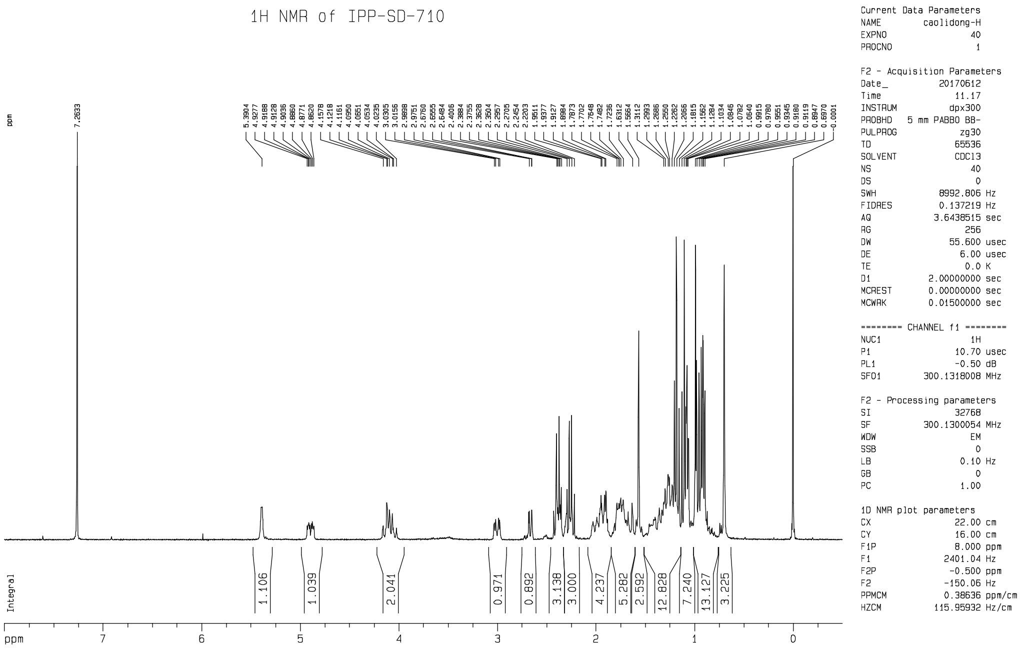

Figure S5. ^{1}H NMR of impurity 1

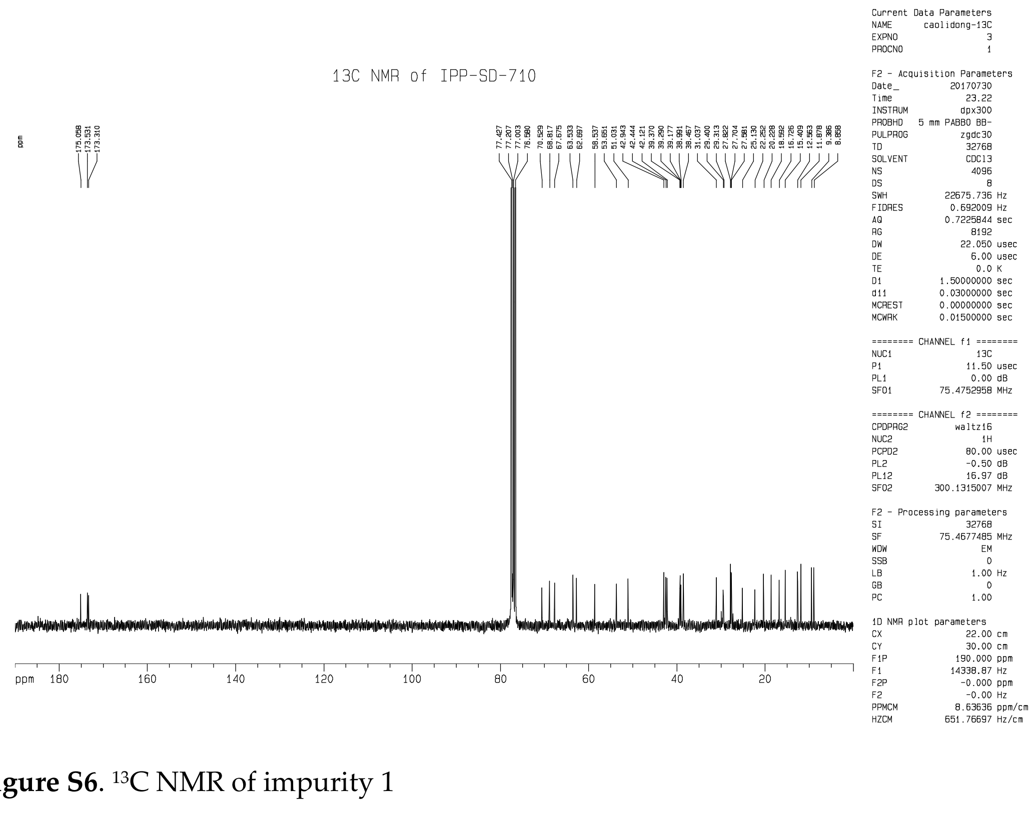

Figure S6. ^{13}C NMR of impurity 1

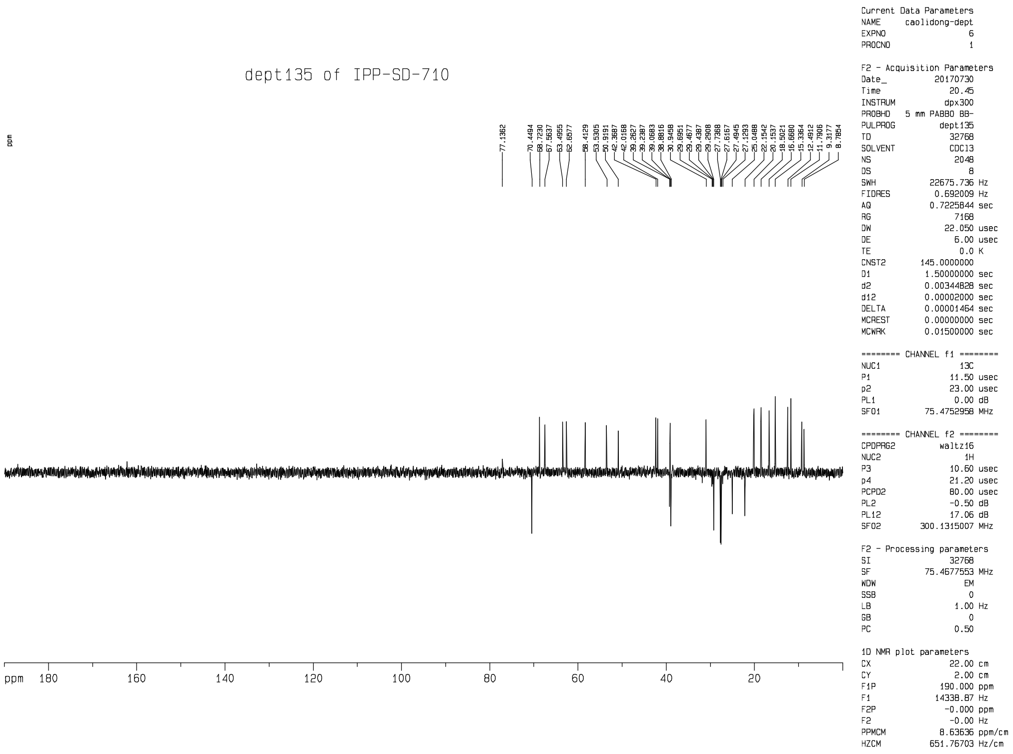

Figure S7. ^{13}C NMR (DEPT 135) of impurity 1

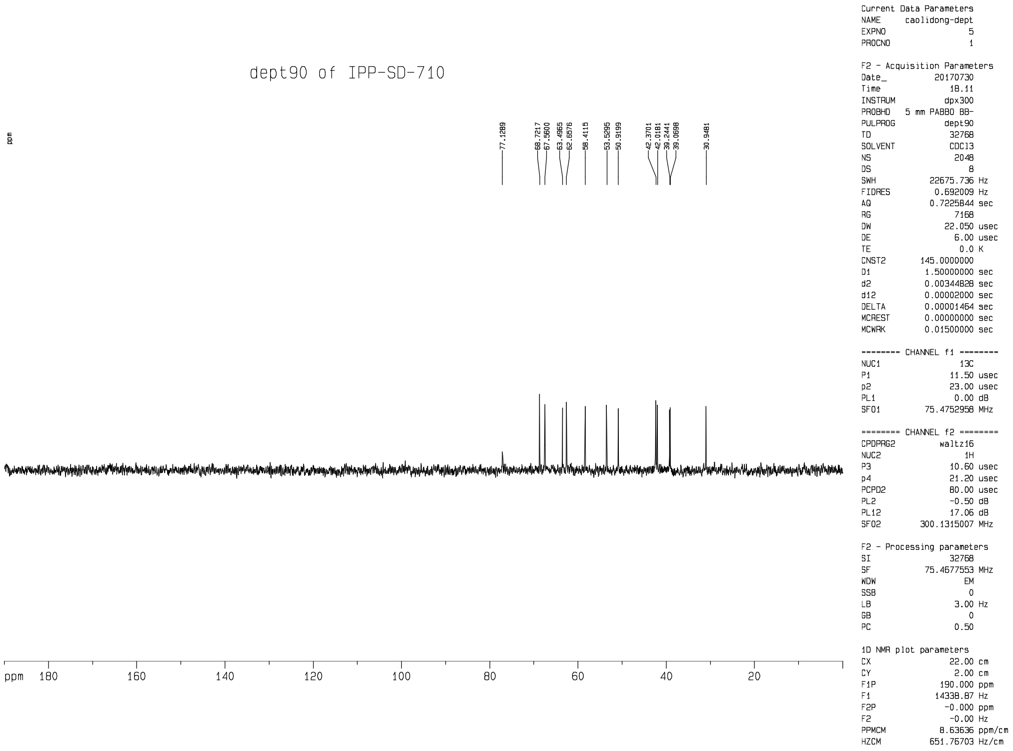

Figure S8. ^{13}C NMR (DEPT 90) of impurity 1

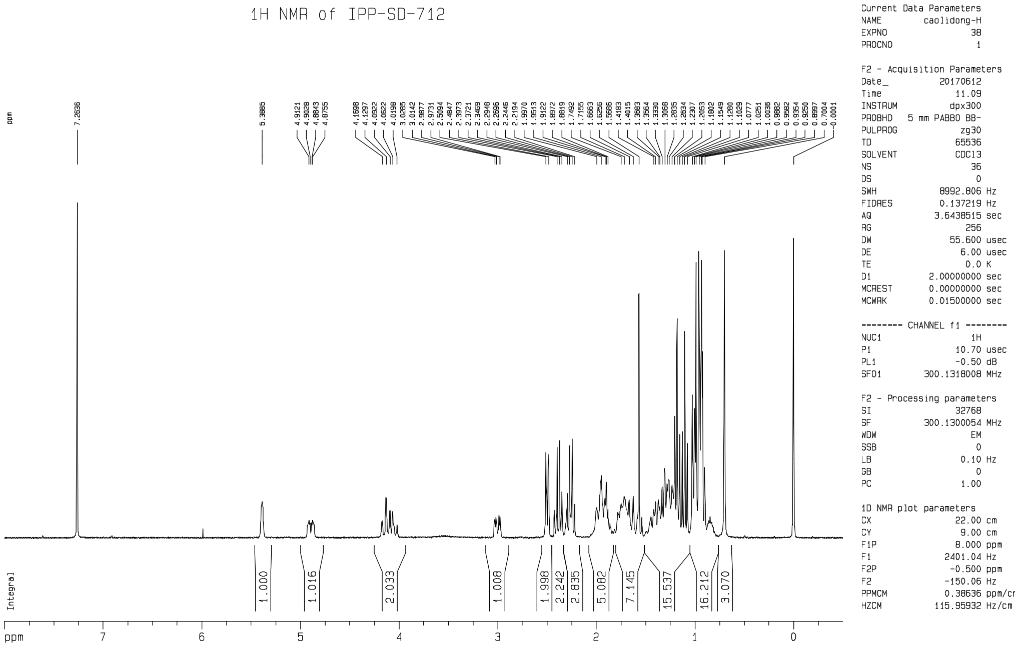

Figure S9. ^{1}H NMR of impurity 2

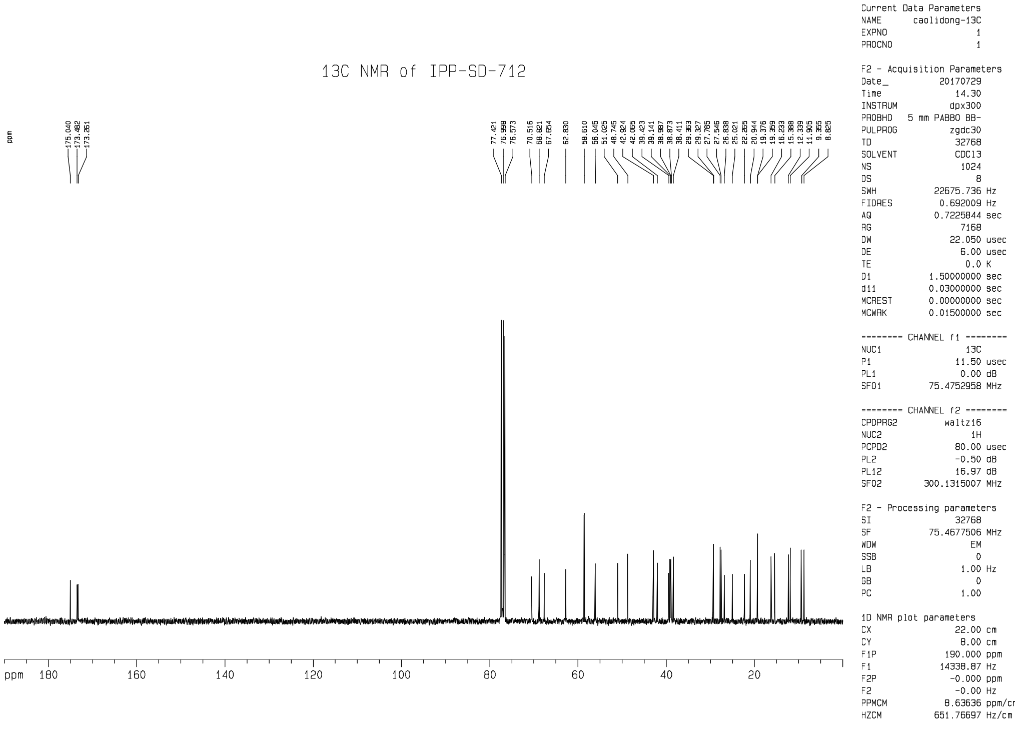

Figure S10. ^{13}C NMR of impurity 2

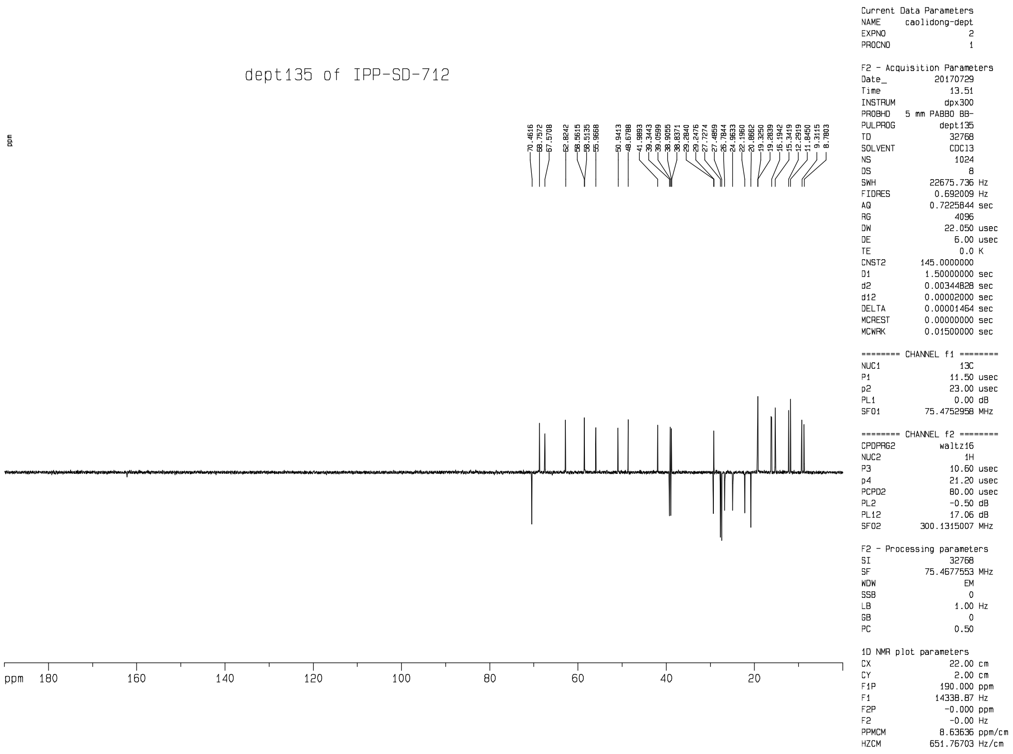

Figure S11. ^{13}C NMR (DEPT 135) of impurity 2

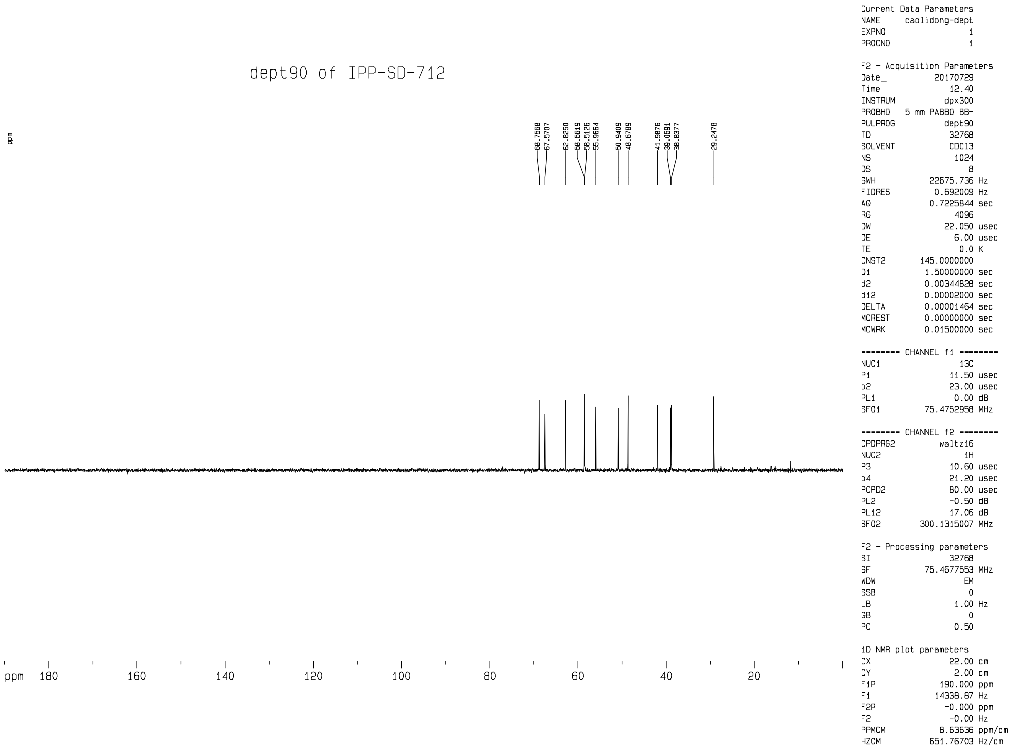

Figure S12. ^{13}C NMR (DEPT 90) of impurity 2

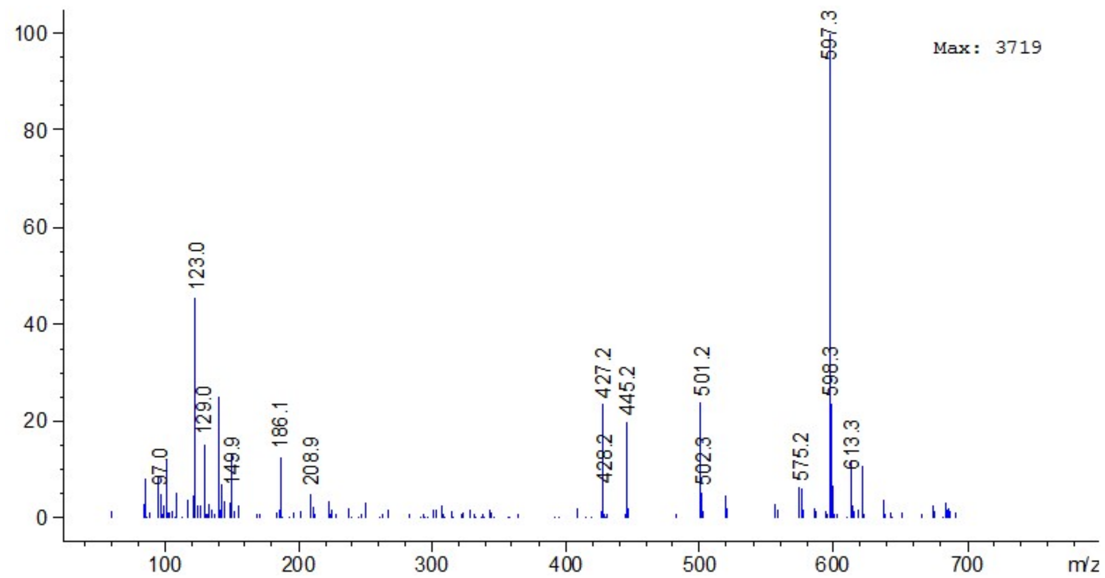

Figure S13. Mass spectrum of propionylbrassinolide impurity 1 recorded in the positive mode.

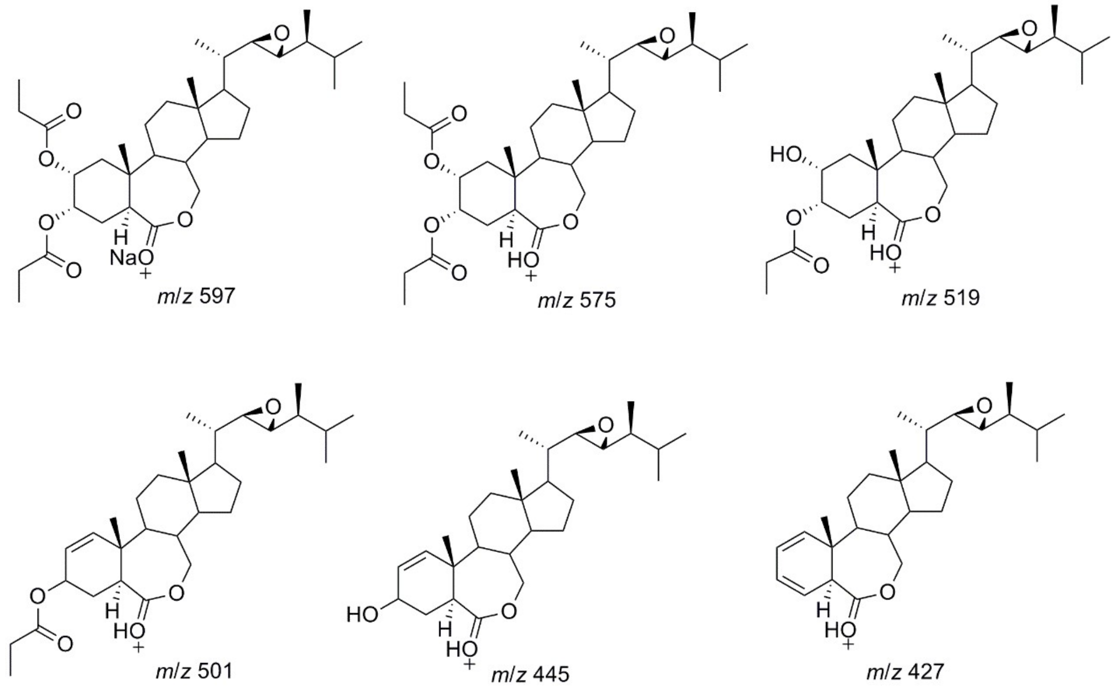

Figure S14. Proposed structure of the major mass ions for propionylbrassinolide impurity 1.

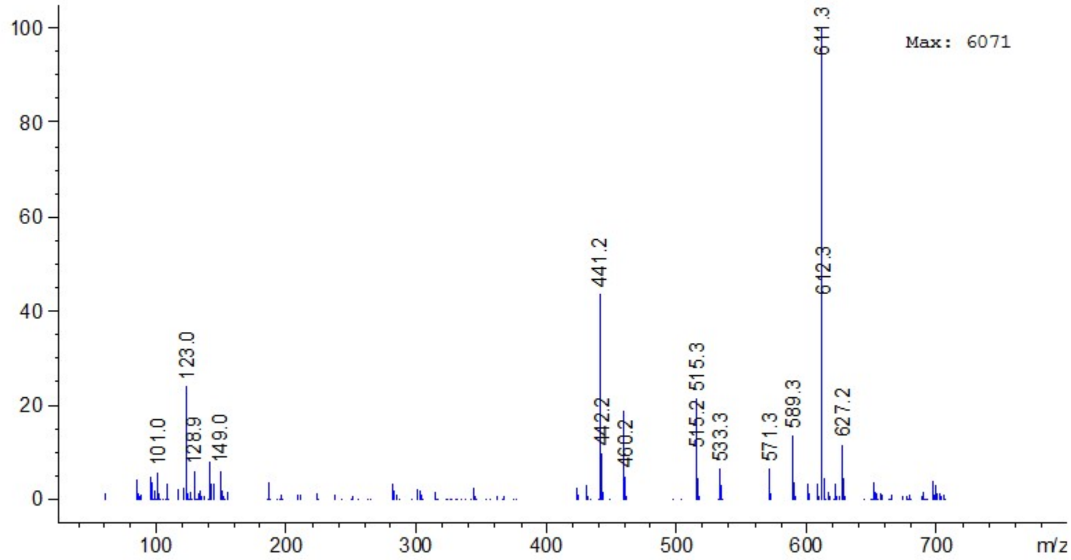

Figure S15. Mass spectrum of propionylbrassinolide impurity 2 recorded in the positive mode.

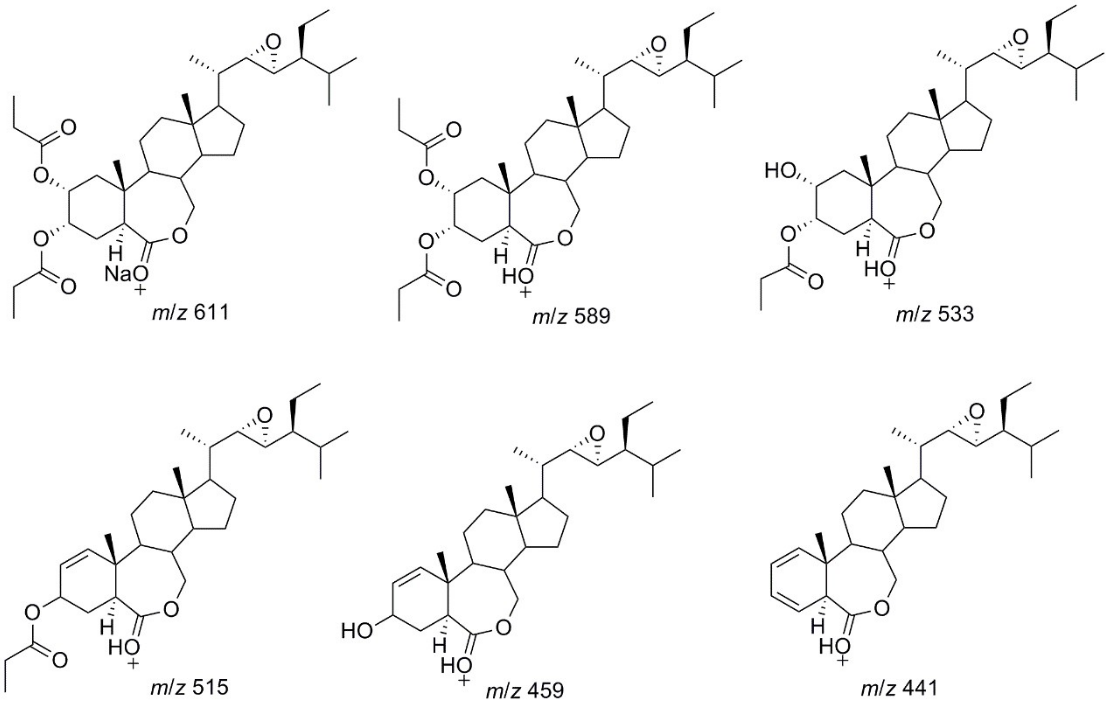

Figure S16. Proposed structure of the major mass ions for propionylbrassinolide impurity 2.
